# Population structure in the Andaman keelback, *Xenochrophis tytleri*: geographical distance and oceanic barriers to dispersal influence genetic divergence on the Andaman archipelago

**DOI:** 10.7717/peerj.5752

**Published:** 2018-10-09

**Authors:** Ashwini Venkatanarayana Mohan, Priyanka Swamy, Kartik Shanker

**Affiliations:** 1 Centre for Ecological Sciences, Indian Institute of Science, Bangalore, India; 2 Department of Ecology and Environmental Sciences, School of Life Sciences, Pondicherry University, Puducherry, India; 3 Dakshin Foundation, Bangalore, India

**Keywords:** Phylogeography, Dispersal, Population genetics, Gene flow, Endemism, Colubridae, Andaman Islands

## Abstract

Limited gene flow between populations due to geographic distance, presence of barriers or inherent low dispersal ability leads to the formation of genetically structured populations. Strong population structure indicates lowered levels or absence of gene flow which might lead to inbreeding and loss of genetic capacity to recuperate from anthropogenic stress and natural calamities. Terrestrial reptiles are generally known to have low dispersal abilities and few studies have explored drivers of their population structure on continental islands, where both anthropogenic stress and natural calamities are relatively common. We investigated the population structure and drivers of diversification of the Andaman keelback (*Xenochrophis tytleri*), an endemic, terrestrial and freshwater snake species in the Andaman archipelago, a continental group of islands in the Bay of Bengal. Data was collected from 86 individuals from seven islands and 78 individuals were sequenced for the gene Nuclear Dehydrogenase subunit 4 to identify the number of populations and distribution of genetic diversity across populations. We found 11 haplotypes on seven islands and observed high genetic differentiation between seven populations defined island-wise (*F*_ST_ = 0.82). We further tested the number of populations by incorporating spatial data into Bayesian Clustering Analysis (GENELAND) and identified six populations of the Andaman keelback. We tested for the influence of Isolation-by-distance on these populations. While the overall trend showed a positive correlation between geographic and genetic distance, a correlogram revealed that the positive correlation disappears beyond ∼20–40 km. We also tested for the presence of geographical barriers to gene flow using Monmonier’s algorithm (SPADS), which identified five barriers to dispersal confirming that there are oceanic barriers to dispersal for some island populations of the Andaman keelback. As the Andaman Islands are arranged almost in a straight line from North to South, our data are insufficient to tease apart the roles of geographical distance and barriers to gene flow. We conclude that salt waters between near islands are weak barriers and as the geographical distance between islands increases, so does the strength of the barrier.

## Introduction

Limited gene flow between populations of organisms due to distance, barriers or low dispersal ability is known to result in genetically structured populations. The insularity of islands and variation in biotic and abiotic conditions makes them ideal natural laboratories to test population biology theories. Islands present some of the most diverse outcomes of evolutionary processes and have therefore been instrumental in understanding evolution and speciation (Losos & Ricklefs, 2009). In the case of terrestrial and freshwater species, populations between islands are expected to be strongly differentiated due to the presence of oceanic barriers (Bottin et al., 2005). On the other hand, populations within islands may lack genetic diversity due to founder effects, small size, isolation and stochastic processes like genetic drift (Carlquist, 1980; Crawford, Stuessy & Silva, 1987; Brauner, Crawford & Stuessy, 1992; Elisens, 1992; Kwon & Morden, 2002).

To explain speciation and genetic variation in space, the concept of isolation-by-distance (IBD) (Wright, 1943) and the role of environmental barriers, such as landscape features in limiting species distribution (Wallace, 1876), have been considered. While landscape features can be strong barriers, the dispersal pattern of a species results from the combined effects of inherent dispersal capacity and inhibiting factors, which in turn has significant implications on evolution and population genetics (Grosberg & Cunningham, 2001; Palumbi, 1994). Low dispersal ability has a higher influence on the population structure with increasing geographical distance, on the other hand, organisms with high dispersal ability experience minimal influence of geographical distance. The limitation imposed by barriers on dispersal could be especially critical for small vertebrates due to their low dispersal capacity (Schippers et al., 1996; Cushman et al., 2006; Wang, Savage & Bradley Shaffer, 2009).

Wright (1931) suggests that large populations with higher migration rates are poorly differentiated when compared to small populations with limited migration, which are highly differentiated. Dispersal of individuals between populations may lead to gene flow which is the key link between ecological traits, local environment and evolution at smaller time scales (Wang, Savage & Bradley Shaffer, 2009). Therefore, studying genetic population structure is critical to understanding the role of a species’ intrinsic factors and other extrinsic factors in their evolution and persistence in habitats undergoing rapid modification. In this study, we focus on the influence of two such extrinsic factors on population genetic structure, distance and barriers.

In general, the dispersal patterns of terrestrial reptiles are poorly understood (Bowne & Bowers, 2004), despite their role as top predators in many ecosystems (Schwaner & Sarre, 1988; Tzika et al., 2008). Terrestrial reptiles on islands could potentially be restricted by oceanic barriers leading to high population differentiation. These populations might, in turn, represent evolutionary significant units, that is, populations historically isolated from each other that have unique genetic potential (Moritz, 1994). Using landscape genetics to evaluate diversity across populations may aid in conserving the evolutionary potential of diverging populations (Grivet et al., 2008). In such intraspecific studies, mitochondrial DNA can serve as a particularly useful tool (Avise et al., 1987; Piganeau, Gardner & Eyre-Walker, 2004) and has been used to detect patterns associated with ecological factors (Richmond & Reeder, 2002; Leaché & Reeder, 2002), understand patterns of variation in morphology (Ashton, 2001), identify important conservation areas (Moritz & Faith, 1998; Mulcahy et al., 2006) and study speciation (Mulcahy, 2008).

The Andaman Islands in the Bay of Bengal are an extension of the Arakan Yoma mountain range; this chain of submarine mountains extends from Cape Negrais of Myanmar to Achin head in Sumatra (Das, 1999). These islands were a continuous landmass during the Late (upper) Pleistocene (Ogg, Ogg & Gradstein, 2016) and the rise in sea level during the Last Glacial Maximum (LGM) has resulted in the isolation of these islands. The Andaman sea is just 200 m deep between all the greater Andaman Islands, Little Andaman and the Ritchie’s archipelago (Smith & Sandwell, 1997). Due to equal depth of the sea between these islands, we assume that the sampled Andaman Islands were last connected to each other at the same time. These islands are a part of the Indo-China biodiversity hotspot and harbor many endemic tropical herpetofauna (Das, 1999). The herpetofaunal affinity of the region with Indo-China is speculated to have resulted from historic land connections during the late Pleistocene glaciations (Das, 1999).

An increasing number of studies have focused on the phylogeography and population genetics of reptiles in South and Southeast Asia; however, intraspecific studies in archipelago systems are lacking. The Andaman keelback (*Xenochrophis tytleri*; Blyth, 1863), classified under the freshwater family Natricidae, is endemic to the Andaman Islands (Vogel & David, 2006). There have been no ecological, behavioral or genetic studies conducted on the Andaman keelback and there is little scientific information about this species despite its common occurrence. It is widely distributed in the archipelago (Vogel & David, 2006) and can be found in and around freshwater bodies and in terrestrial habitats with high moisture content. The Andaman keelback has been sighted close to brackish water bodies or in areas which has brackish water only during high tide, but never in brackish water, which is very unlike several other species of the genus *Xenochrophis* across South and South-east Asia. Absence of Andaman keelback in brackish water could be a case of false-negative, however, lack of proof until now forces us to consider this species as exclusively freshwater.

We hypothesize that the rise in sea level and formation of insular islands during the late-Pleistocene reduced geneflow between the newly restricted subpopulations. Therefore, in this study, we aimed to (i) identify the number of populations of the Andaman keelback, (ii) estimate the genetic connectivity between populations and (iii) understand the effect of IBD and barriers to geneflow between populations.

## Materials and Methods

### Study area and sampling

**Figure 1:**
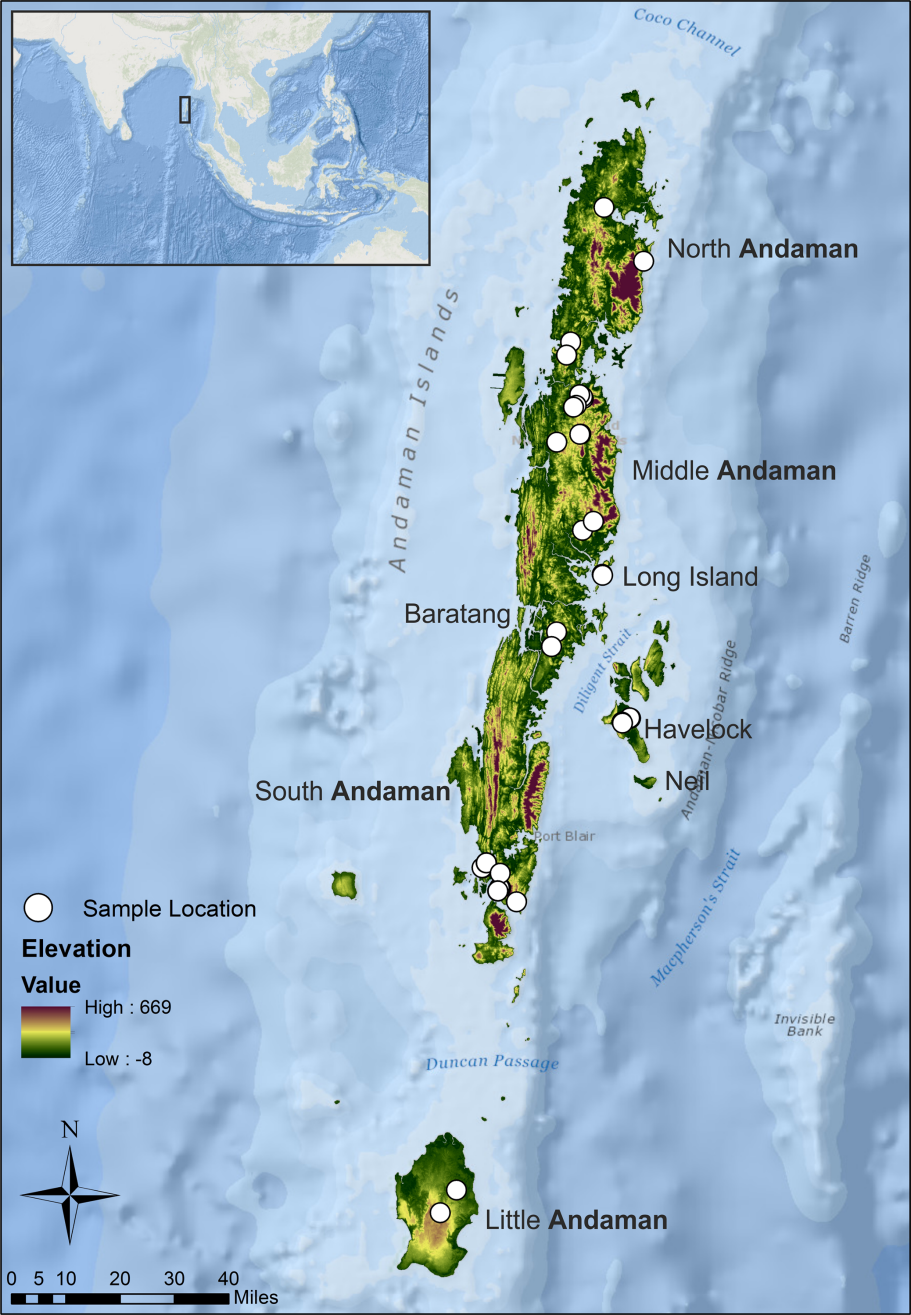
Study area map showing sampling locations from seven islands of the Andaman archipelago. Sampling localities are indicated with white points. Islands with sampling have been labeled by name.

The Andaman archipelago, stretching north to south between 13.66°N, 93.00°E and 10.54°N, 92.46°E, receives annual rainfall of 3,000–3,500 mm (Andrews, Sankaran & Sankaran, 2002). We sampled the Andaman keelback in eight major islands of the archipelago (Fig. 1) from December 2014 to February 2015, but failed to locate any snakes on Neil island. Permission to conduct field surveys, including tissue collection from the Andaman keelback (listed under Schedule IV of the Indian Wildlife (protection) Act 1972) was obtained from the Department of Environment and Forests, Andaman and Nicobar Islands (Permit No. CWLW/WL/134/366). The permit from the Department of Environment and Forests covered all aspects of our study, including field survey, capture and tail tip tissue collection. Our study was limited to the relatively dry period of the year. We conducted sampling in streams within evergreen, semi-evergreen and mixed forests, as well as in human modified areas such as agricultural fields and plantations. The sampling sites were distributed throughout the major islands excluding the Jarawa tribal reserve. The reserve, home to the Jarawa tribe, spans just over 1,000 km^2^ including areas in South and Middle Andaman Islands (Sekhsaria & Pandya, 2010) and access is completely restricted by the government. Due to its large area and location (northern parts of the South Andaman and southern parts of the Middle Andaman), lack of samples from this area do lead to some loss of information about the connectivity of the South Andaman with the rest of the Andaman Islands, especially Baratang. The elevation in sampled points ranged from ca. 1 to 75 m above sea level.

A team of two personnel conducted opportunistic visual encounter surveys between 18:00 and 23:00 h. We spent a total of ca. 240 man hours in locating snakes. Upon locating and capturing the Andaman keelback, we recorded the Global Positioning System (GPS) location (using a GPS Trimble Juno 3) with an accuracy of 2–5 m. When individuals were found on stream banks and wet soil, we captured them with the help of a small stainless-steel hook and bagged them into breathable cotton bags. When individuals were in water, we lifted them from water with both hands and then used the hook to hold them. Captured snakes were restricted in a transparent restraining tube (both ends open). We ensured that the sample collection took minimum time and always provided body balance to individuals. This handling protocol was approved by the Department of Environment and Forest, Andaman and Nicobar Islands Research Advisory Committee and adheres to international Animal Care and Use guidelines.

We determined the sex of each individual using sterilized and lubricated sexing probes (Pro ball tip probe, Midwest Tongs). We used sterilized surgical steel scissors to cut the tail tip (up to 15 mm from the tip). The snakes were not anesthetized while the tail tip was removed. We stored the tissue sample in 1.5 ml vials filled with molecular grade ethanol. After collecting the tissue, we sterilized the open wound and allowed the individual to settle for a few minutes before releasing them at the capture site. The time taken in handling individuals did not exceed 10 min. The tissue samples were deposited at the Centre for Ecological Sciences, Indian Institute of Science, Bangalore (CESS 1600-CESS 1686). Funds were not available for post release monitoring of sampled individuals. However, individuals of the Andaman keelback sampled in our research base station, Andaman and Nicobar Environmental Team (ANET), a long-term monitoring site, showed no signs of side effects after sampling. These individuals could be identified by their excised tail tip and individuals were frequently sighted in the same areas they were found in prior to sampling.

### Mitochondrial gene sequencing

We extracted genomic DNA from 86 Andaman keelback individuals from 38 locations (3–27 individuals from each island) using the phenol chloroform method (Sambrook, Fritsch & Maniatis, 1989). To test if the mitochondrial genome showed diversity between individuals sampled from different islands, we sequenced the 16S rRNA (16S, 423 bp) coding gene from 16 and Cytochrome B oxidase gene (CytB, 302 bp) from 12 representative samples from seven islands. For six samples, we obtained both CytB and 16S data. After evaluating pairwise genetic distances between these individuals, we proceeded with further analyses to test population differentiation. We then sequenced a relatively fast evolving gene, Nuclear Dehydrogenase subunit 4 (ND4, 560 bp) gene for all the samples collected, from which 78 samples produced good quality sequences. Amplification of 16S was done using 16S SAR and 16S SBR primers (Simon et al., 1994), ND4 using ND4 and LEU (Arèvalo, Davis & Sites, 1994) and Cytochrome B using H16064 (Burbrink, Lawson & Slowinski, 2000) and L14910 (De Queiroz, Lawson & Lemos-Espinal, 2002). We amplified the DNA for 35 cycles with an annealing temperature of 46 °C for 16S region, 53.3 °C for ND4 region and 47 °C for CytB region. We then purified the amplified DNA using QIAquick PCR purification kit. Sequencing was carried out using the Sanger (Dideoxyribonucleotide chain termination) method (Sanger, Nicklen & Coulson, 1977). Cytochrome B and ND4 sequences were translated into amino acid sequences and corrected for reading frame, to ensure that no stop codons were present in the sequences. All sequences used in this study were deposited in GenBank (Table S1).

### Data analysis

#### Genetic diversity and differentiation

Before aligning the sequences, we checked them manually to ensure that ambiguous sites were excluded from further analyses. We aligned the sequences using Muscle (Edgar, 2004) with default parameters set in MEGA5.2 (Tamura et al., 2011). To understand genetic variation, we estimated the number of haplotypes (h) and haplotype diversity (H_d_) in DnaSP 5.10.01 (Librado & Rozas, 2009). We constructed the haplotype network using NETWORK 5.0 (fluxus-engineering.com) using the Median-joining method (Bandelt, Forster & Röhl, 1999), and subsequently modified it to include details on geographical location. We constructed Median Joining Haplotype networks from the 16S and CytB dataset to realize their haplotype diversity, but all further analyses were carried out using the ND4 gene dataset. To test whether the ND4 gene was under selection, we calculated Tajima’s D in the software Arlequin 3.5.2 (Excoffier & Lischer, 2010).

We used hierarchical partitioning of molecular variance (AMOVA) to assess the level of population subdivision and obtained population pair wise *F*_ST_ using DNA sequence divergence and allele frequency in Arlequin 3.5.2. We investigated population subdivisions at two levels; populations and groups (Table S2). Individuals were assigned a priori to populations based on the island of their origin and populations in turn were assigned to groups based on the geographic position of the islands. Grouping island populations into northern and southern groups yielded the highest *F*_ST_ values; the northern group consisted of populations from North Andaman, Middle Andaman, Baratang, Long island and Havelock and the southern group consisted of populations from South and Little Andaman.

#### Drivers of diversification

To examine the effect of geographical distance on observed genetic distances, we performed Mantel test in R using the package Vegan, Permute and Lattice (R Core Team 2016; Oksanen et al., 2018). We constructed scatter plots to evaluate relationships between individual pairwise genetic and geographical distances. Later, after identifying the number of populations in GENELAND, we constructed scatter plots with island population pairwise *F*_ST_ and shortest geographical distance between a pair of populations. This was calculated by evaluating Tamura-Nei pairwise genetic distances between all individuals belonging to different populations using MEGA 7.0 (Kumar, Stecher & Tamura, 2016). Straight line geographical distances between all sampled points were obtained using Quantum GIS (QGIS, 2011). We calculated the geographical distances using Geographic Distance Matrix Generator (Ersts, 2011). For the population pairwise *F*_ST_ calculation, we used the values from the “best run” previously used to assess the level of population subdivision. Overall correlation does not yield details of a relationship across a distance range; therefore, we examined the correlation at specific distance intervals by plotting a correlogram. Distance classes defined to construct the correlogram were 20, 60, 100, 160 and 320 km.

#### Spatial genetic analysis

We assessed geographical structure and the number of populations using GENELAND with the Markov Chain Monte Carlo (MCMC) method (Guillot, Mortier & Estoup, 2005). We performed ten replicate runs with 2 × 10^5^ MCMC iterations with a thinning value of 10. Spatial coordinates had no uncertainty and a maximum rate of Poisson process fixed to 100. The number of populations and population membership from both correlated and uncorrelated models were analyzed and produced similar results. We tested the number of population clusters (K) from 2 to 14; the number of identified clusters saturated at 6. We then fixed K at 6 to estimate the other parameters included in the model, including number of populations, population membership probability of individuals, map of population membership and posterior density of the model.

We subjected the data to AMOVA by assigning individuals to populations obtained in GENELAND and groups were not changed (Northern group: North Andamans, Mayabunder, Rangat and Baratang; Southern group: South and Little Andamans). To test whether areas of cluster transition observed in the map of population membership was an artefact of clustering, we tested for the presence of barriers. We determined the barriers by employing Monmonier’s maximum difference algorithm (Monmonier, 1973) in the program SPADS (Spatial and Population Analysis of DNA sequences) (Dellicour & Mardulyn, 2014). Monmonier’s maximum difference algorithm recognizes areas associated with highest rate of change between sampled populations on a Delaunay triangulation network connecting all sampled populations (Manni, Guerard & Heyer, 2004). For this analysis, we first considered each island as a single population. We used geographic data consisting of GPS points of one representative sample from each population (the same sample used to determine shortest geographical distance for correlation tests) and population pairwise *F*_ST_ generated in Arlequin 3.5.2. Single locus average *F*_ST_ estimator computed using Monmonier’s algorithm in SPADS for both the analyses were visualized in the software Barriers (Manni, Guerard & Heyer, 2004). We repeated the analysis by specifying a different upper limit for the number of barriers, ranging from 10 to five with reduction of one barrier in each run. The barriers detected did not increase beyond five (unique barriers) separating six populations. To test whether barriers identified for GENELAND classified populations would be different from island populations, we generated population pairwise *F*_ST_ for GENELAND populations in the software Arlequin 3.5.2 and followed the same method to specify geographical points for each population and identify barriers. The “maximum number of barriers” was set at six for seven island populations and five for six GENELAND identified clusters to demonstrate that the same five barriers were detected irrespective of the value set for this parameter.

## Results

### Genetic diversity and differentiation

**Figure 2:**
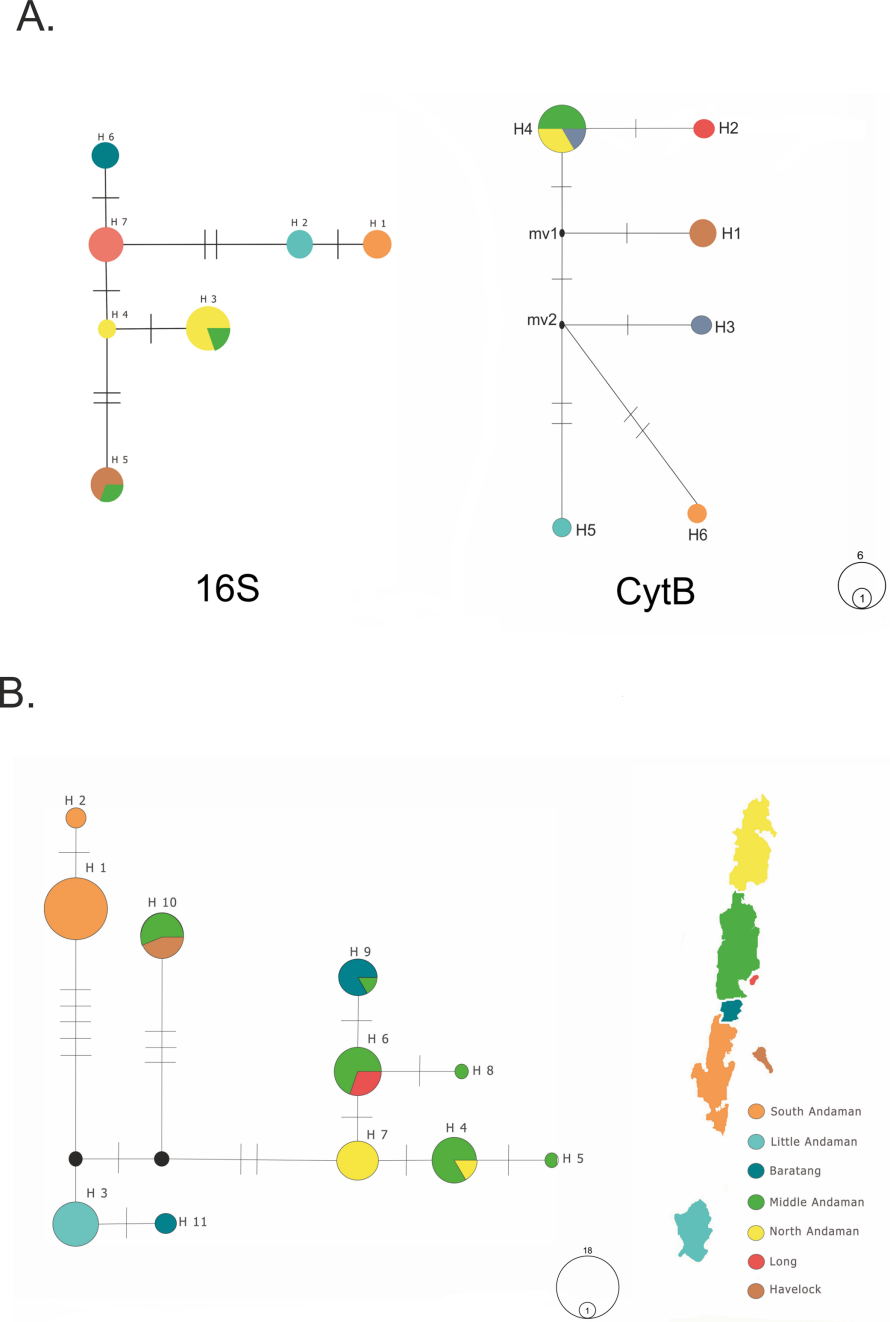
Median-joining haplotype networks. (A) Median-joining haplotype network from *16S* and *CytB* gene sequences. H 1–H 7: labels of each haplotype. Number of ticks on the network between haplotypes correspond to number of base pair differences between them. (B) Median-joining haplotype network from *ND4* gene sequences. H 1–H 11: labels of each haplotype, number of ticks on the network between haplotypes correspond to number of base pair differences. Filled black circles correspond to median vectors. The circle size of each haplotype corresponds to the haplotype frequency.

We obtained seven haplotypes of 16S (*H*_d_ = 0.86) with a total of seven variable positions and six haplotypes from CytB (*H*_d_ = 0.75) with a total of nine variable sites (Fig. 2A). Pairwise genetic distances from the maximum composite likelihood method ranged from 0 to 0.007 for 16S and 0.00 to 0.017 for CytB. From the ND4 gene sequences from 78 samples, we identified 11 haplotypes with a haplotype diversity *H*_d_ = 0.87, nuclear diversity π = 0.009 and 18 variable positions (Fig. 2B). The outcome from Tajima’s test (*D* = 1.25, *p* > 0.10), although positive, was statistically not significant. Pairwise genetic distances ranged from 0 to 0.02. The gene ND4 has unique haplotypes in most islands, but there are also common haplotypes shared between islands (Fig. 2B). Middle Andaman Island has six different haplotypes, two of them unique to the island and four others shared with four different islands and vice versa. On the other hand, both Little Andaman and South Andaman do not share haplotypes with any other islands.

Populations differed significantly in their genetic composition (Table 1) when tested using hierarchical AMOVA. High genetic differentiation among groups among populations (*F*_ST_) and among populations within groups (*F*_SC_) were obtained (Table 1).

**Table 1:** Analysis of molecular variance (AMOVA) for seven populations of the Andaman keelback on the Andaman archipelago.

Source of variation	VC^a^	PV^b^ (%)	*p*	Fixation indices
Among groups	1.73	45.96	0.11	*F*_CT_ = 0.46
Among populations within groups	1.38	36.29	<0.001*	*F*_SC_ = 0.69
Among populations among groups	0.68	17.74	<0.001*	*F*_ST_ = 0.82

**Notes:**

Population classification: North Andaman, Middle Andaman, Baratang, South Andaman, Little Andaman, Havelock and Long Island were considered as individual populations.

a VC, variance,

b PV, percentage variation.

* Indicates statistical significance, statistical significance was set at α = 0.05.

### Drivers of diversification

**Figure 3:**
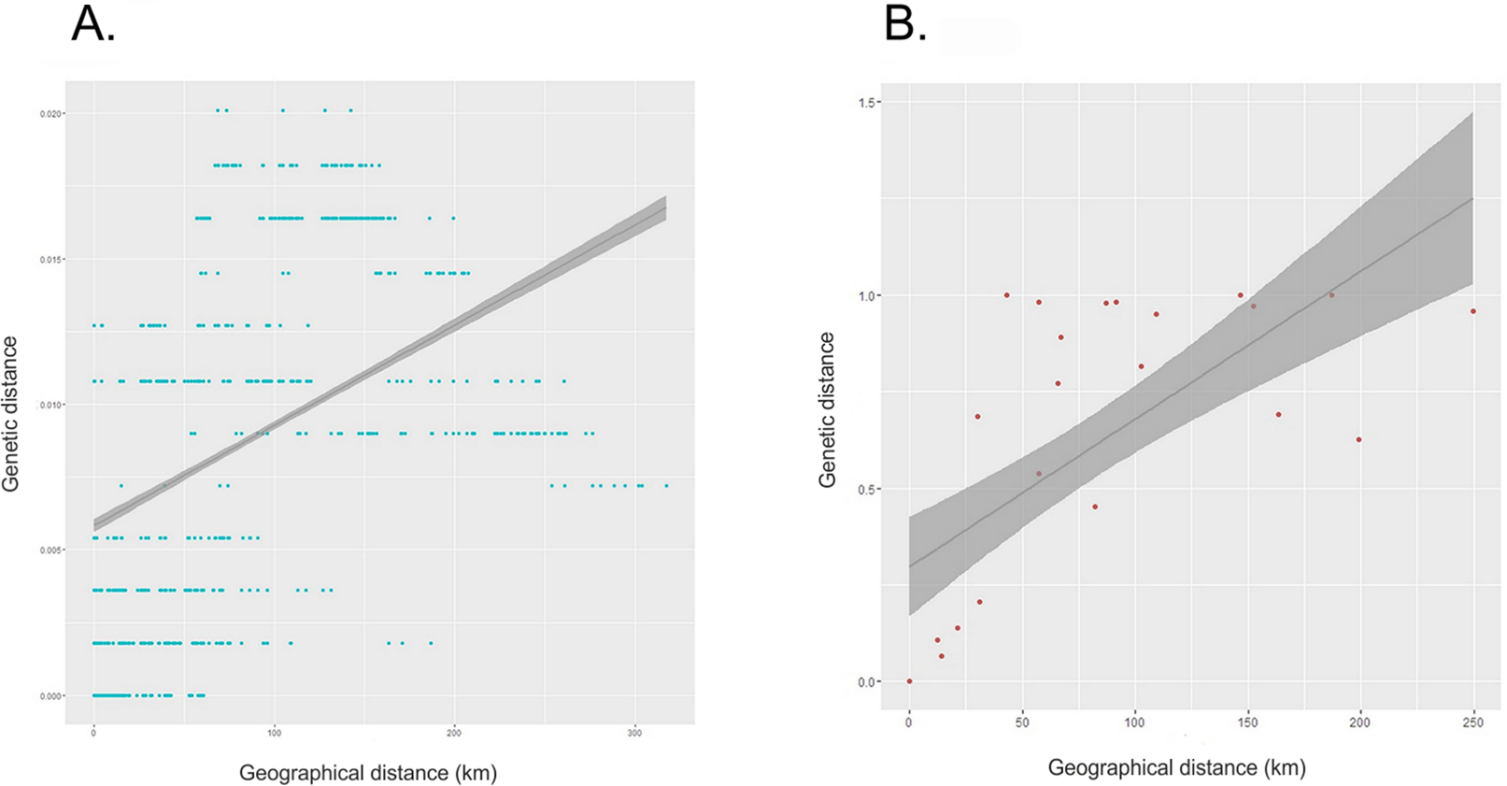
Correlation between genetic and geographic distances. (A) Scatter plot depicting the relationship between individual pairwise genetic distances (Tamura-Nei distance) and pairwise geographical distances; (B) Scatter plot depicting the relationship between population pairwise genetic distances (*F*_ST_) and pairwise geographical distances.

**Figure 4:**
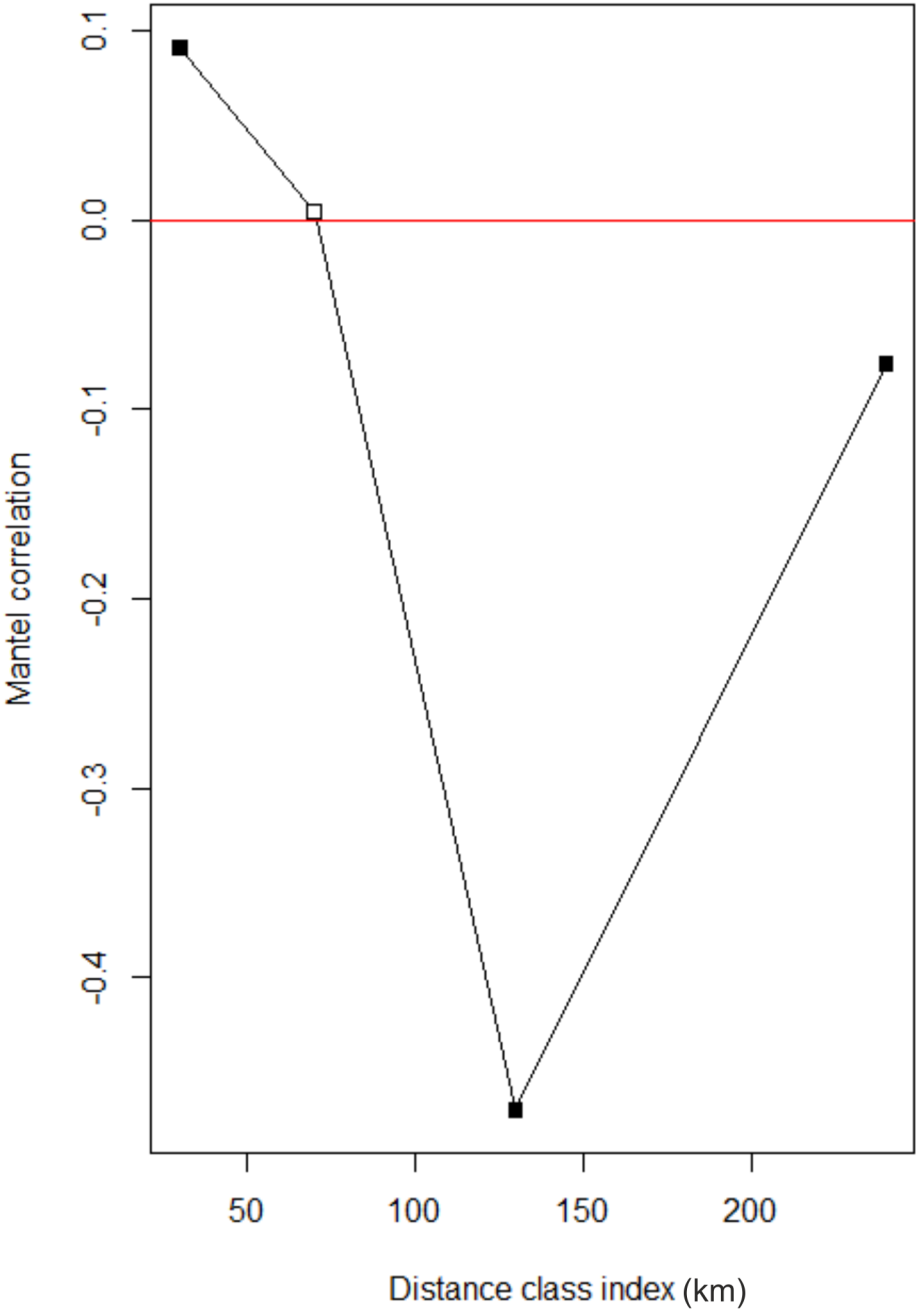
Mantel correlogram for individual pair wise genetic distance vs. individual pair wise geographical distances. All correlations are significant at α = 0.05 with Bonferroni-corrected *p*-values.

Both individual and population genetic distances were positively associated with geographical distances (individuals: *r* = 0.401, *p* = 0.001; populations: *r* = 0.550, *p* = 0.027). Individual genetic and geographical distances showed a weak positive correlation (*R*^2^ = 0.181, β = 5264.386, SE = 143.507, *p* < 0.001) (Fig. 3A), but the correlation between population pairwise genetic distances and geographical distances (*R*^2^ = 0.448, β = 120.157, SE = 19.032, *p* < 0.001) explained almost half the variance (Fig. 3B). In the correlogram, association of individual genetic distances with geographical distances at 20–60 km distance interval showed high, significant positive correlation but at 100 km intervals, we observed a significant negative correlation (*r* = −0.4 *p* = 0.003) (Fig. 4 and Table S3).

### Spatial genetic analysis

**Figure 5:**
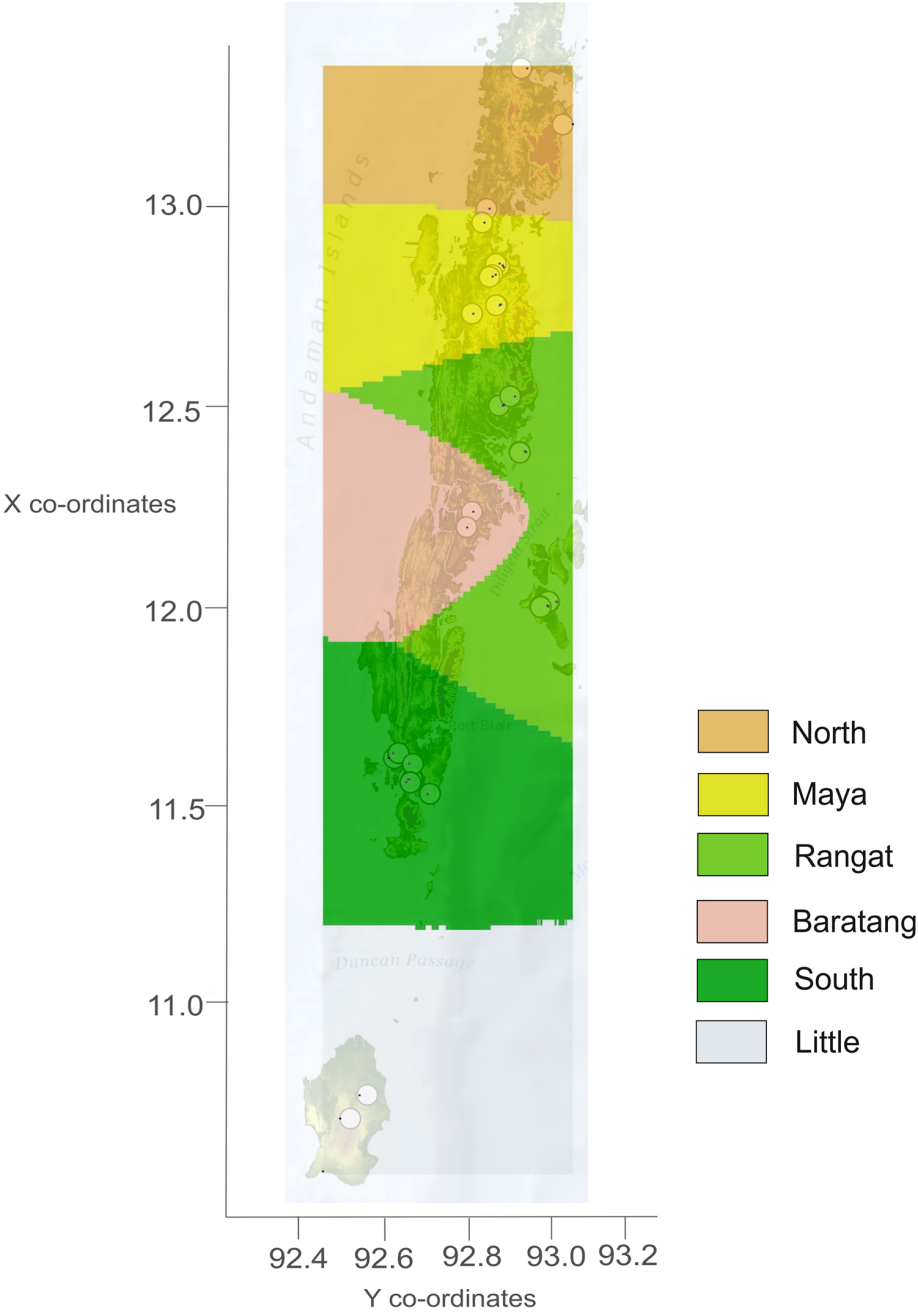
Map of population membership of individuals obtained from GENELAND. Cluster membership result from GENELAND overlaid with the map of Andaman Islands in CorelDraw X7 (Map not to scale).

**Figure 6:**
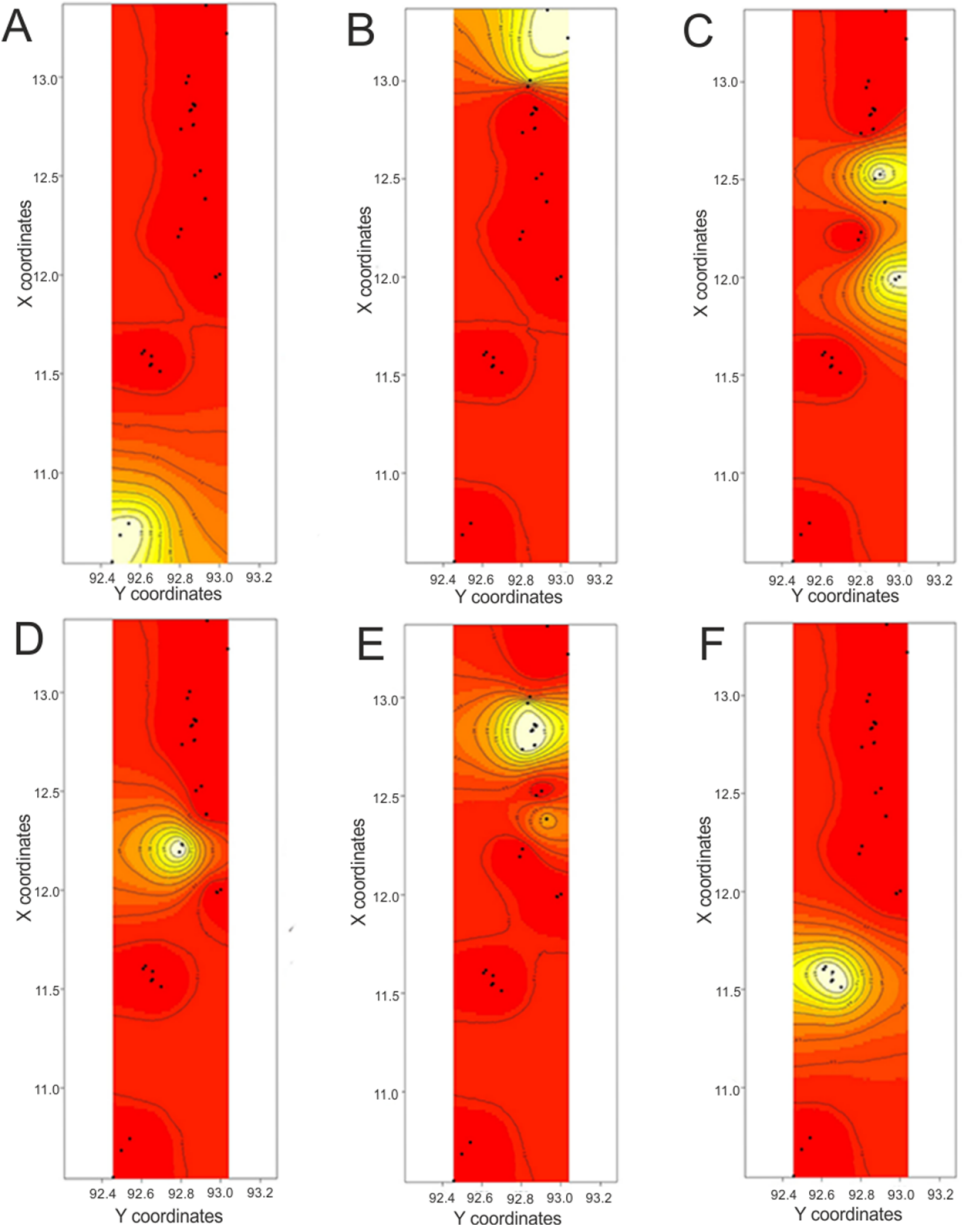
Maps of posterior probability of individuals to belong to clusters. Sampled locations appear as black points on the maps. Maps are color coded based on posterior probability values–red, low probability (0.1) to white, high probability (1.0). (A) Map of posterior probability of individuals to belong to cluster Little Andaman, (B) Map of posterior probability of individuals to belong to cluster North Andaman; (C) Map of posterior probability of individuals to belong to cluster Rangat, (D) Map of posterior probability of individuals to belong to cluster Baratang; (E) Map of posterior probability of individuals to belong to cluster Mayabunder, (F) Map of posterior probability of individuals to belong to cluster South Andaman.

The Bayesian clustering algorithm in GENELAND identified six clusters from seven islands. After fixing the number of population clusters (*K* = 6), we obtained a map of population membership (Fig. 5). The populations include (1) Little Andaman Island (hereafter Little Andaman), (2) South Andaman Island (South Andaman), (3) Baratang Island (Baratang), (4) southern parts of Middle Andaman Island and Havelock Island (Rangat), (5) northern parts of Middle Andaman and Long Island (Mayabunder) and (6) North Andaman Island (North Andaman). The maps of posterior probability that individuals belong to clusters (Fig. 6) all show high probability (≥0.9). The results from AMOVA with populations defined from GENELAND produced a higher *F*_ST_ value (Table 2) than results from AMOVA with seven populations defined a priori (Table 1).

**Table 2:** Analysis of molecular variance (AMOVA) for six populations of the Andaman keelback classified in GENELAND.

Source of variation	VC^a^	PV^b^ (%)	*p*	Fixation indices
Among groups	1.69	44.98	0.06*	*F*_CT_ = 0.45
Among populations within groups	1.53	40.62	0*	*F*_SC_ = 0.74
Among populations among groups	0.54	14.40	0*	*F*_ST_ = 0.86

**Notes:**

Population classification: North Andaman, Mayabunder, Rangat, South Andaman, Little Andaman, Baratang.

a VC, variance,

b PV, percentage variation.

* Indicates statistical significance, statistical significance was set at α = 0.05.

**Figure 7:**
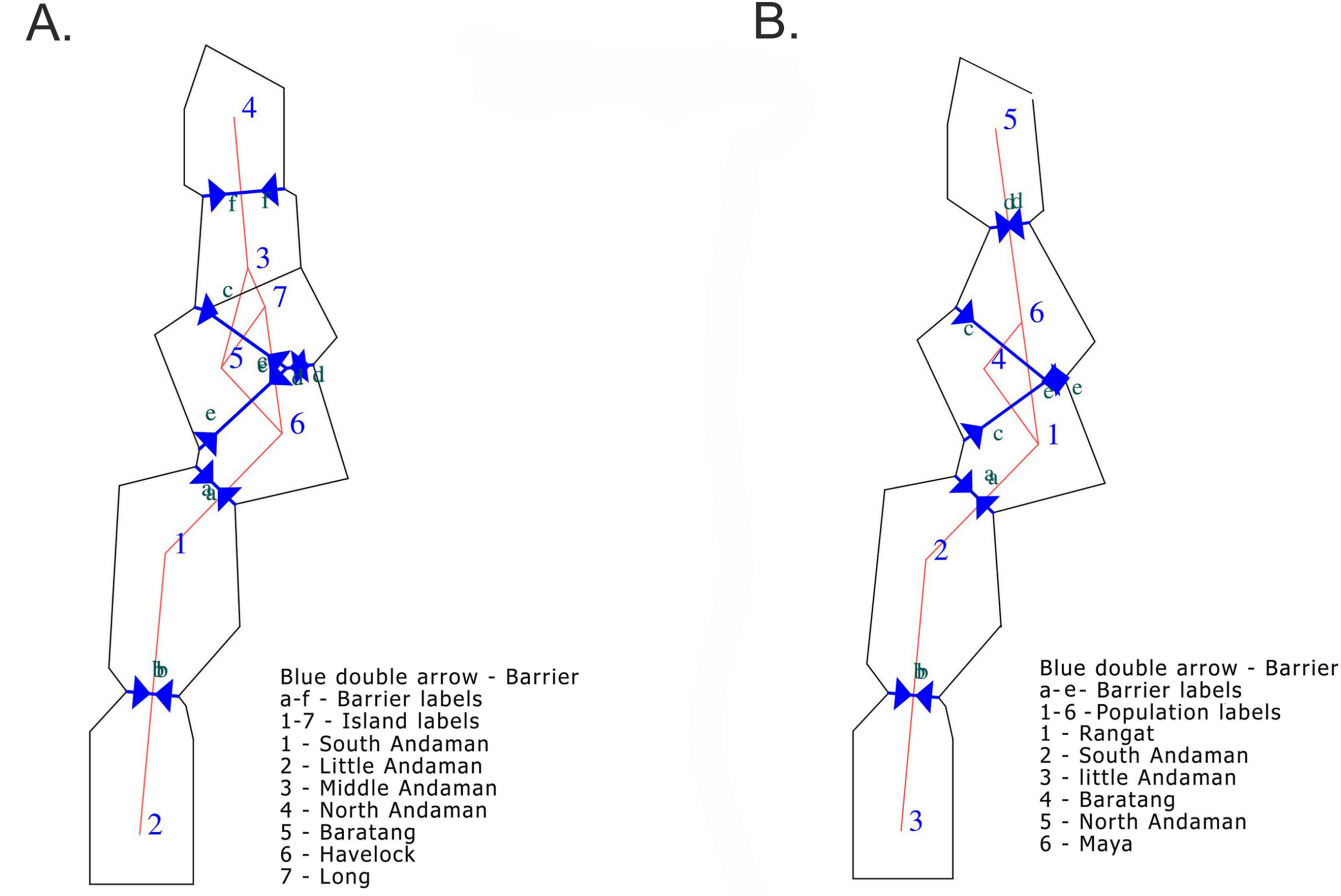
Geographical barriers between Island populations and GENELAND populations of the Andaman Keelback. (A) Barriers detected between populations from seven islands-North Andaman, Middle Andaman, Baratang, South Andaman, Little Andaman, Havelock and Long Island; upper limit for the number of barriers was set to six. (B) Barriers detected between populations identified in GENELAND-North, Mayabunder, South, Baratang, Rangat and Little; upper limit for the number of barriers was set to five.

We identified five potential barriers to the dispersal of the Andaman keelback between seven island populations (Fig. 7). These barriers led to the identification of six populations from seven islands, which is concordant with the results from GENELAND. Although the number of populations and individuals assigned to populations differed between the two analyses for barrier detection, the barriers identified were the same. Barring two barriers (f, d: A and d, e: B, Fig. 7), all inferred barriers were salt-water barriers between islands.

## Discussion

We detected high diversity of mitochondrial haplotypes between populations from different Andaman Islands, however, their diversity within islands were not high except Middle Andaman Island. We identified six populations of the Andaman keelback from seven major islands of the Andaman archipelago (Fig. 6). All populations, barring two, are physically separated by salt water. Our study supports the hypothesis that salt water serves as a barrier to gene flow between populations of the Andaman keelback, but does not fully explain their population structure. We discuss the role of geographic distance, barriers to dispersal and other factors in shaping the genetic diversity and distribution in populations of the Andaman keelback snake.

### Genetic diversity and differentiation

The highest genetic diversity in the ND4 gene was detected on Middle Andaman Island which could be due to its central location and its large area (1,536 km^2^). We detected six different haplotypes, two of them unique to the island and four others shared with four different islands namely Havelock, Long Island, Baratang and North Andaman Island. South and North Andaman Islands are about the same size and could have more haplotypes which were not detected due to insufficient sampling. This study detected both shared and unique haplotypes in both 16S and CytB markers, but this is based on very few samples, so no inferences can be made.

Our results on the haplotype distribution are comparable to a phylogeography study on Bat populations on the Andaman Islands; Middle Andaman Island houses maximum haplotype diversity and shared haplotypes with surrounding smaller islands reflecting the larger area and a potential source population (Chakravarty et al., 2018).

We observed statistically significant, high levels of differentiation among groups among populations (*F*_ST_) and among populations within groups (*F*_SC_) (Table 1). This reflects the limited overlap of haplotypes between populations. In the Cycladic Archipelago in the Aegean Sea, Aegean wall lizard’s populations show little or no contemporary gene flow between different island populations (Santonastaso et al., 2017). This is a group of continental islands comparable to the Andaman Islands as they were also formed due to rise in sea levels in the LGM. In general, gene flow appears to be restricted due to ocean barriers in island systems (Wilson & MacArthur, 1967).

Each sampled population of the Andaman keelback displayed low genetic diversity, and were highly differentiated due to limited gene flow and dispersal. As we detected no selection for alleles of ND4 gene in these populations, the genetic differentiation observed among populations is likely to be purely a result of genetic drift.

### Drivers of diversification

Observed positive correlations between genetic and geographical distances would appear to indicate that IBD is driving diversity patterns (Fig. 3). However, IBD does not capture the role of complex landscapes and historical processes (Wilson & MacArthur, 1967). A careful consideration of the study area reveals that with an increase in geographical distance, the number of barriers also increases. The Andaman Islands are arranged in a linear manner which can be attributed to their geological history (Das, 1999). Therefore, the observed positive correlation may not only be due to IBD, but the result of strong barriers to dispersal or a combination of both these factors. From the correlogram, we observe a strong positive correlation between genetic and geographical distances at a distance class of 20 km; but as the geographical distance increases, there is a decrease in correlation (Fig. 4). This suggests that, beyond barriers, the effect of IBD is low. If IBD is a strong driver of genetic diversity, this correlation should remain positive at all distance classes. It seems more likely, therefore, that IBD may drive genetic differentiation up to a certain short distance or in other words, within an island, but beyond the island, salt water has a higher influence on phylogeographic patterns of the Andaman keelback. Considering the natural history of this species, short stretches of salt water between islands could function as strong barriers to geneflow, but this can only be confirmed through additional sampling close to boundary areas of Middle Andaman, Baratang and South Andaman Islands. On the other hand, Middle and North Andaman share a haplotype despite presence of a short stretch of sea water, but our data is not sufficient to tease apart the roles of short stretch of sea water and/or construction of a road bridge in detection of this haplotype.

### Spatial genetic patterns

Populations of the Andaman keelback identified using both genetic and spatial data assigned individuals to six population clusters (Fig. 6) reflecting high levels of population differentiation, mirroring the results of AMOVA (Table 1). The AMOVA utilizing populations identified by GENELAND led to very similar values of population differentiation. This indicates strong population differentiation and limited gene flow between most islands. Islands of Little Andaman, South Andaman and Baratang have exclusive populations of the Andaman keelback. Middle Andaman Island has two populations, namely the northern population (Mayabunder) which also includes individuals from Long Island and a location from the North Andaman, and the southern population (Rangat) includes individuals from Havelock Island. The partitioning of individuals from the Middle Andaman into two populations could be due to insufficient sampling in central parts of Middle Andaman. We were unable to obtain any individuals from this region as we sampled during relatively dry periods. The North Andaman Island (except the southernmost sampled location) has a single population of the Andaman keelback. There could be gene flow between these two populations; however, it is important to note that these two islands have been connected by a bridge for the last ∼20 years which facilitates dispersal of amphibians and small terrestrial reptiles during the monsoon (A. V. Mohan, 2014, personal observation).

Inference about gene flow patterns between two populations can be made easily in the presence of sharp geographic boundaries between them (Jenkins et al., 2010). We identified a few additional barriers to dispersal beyond those obtained from populations based on GENELAND. All barriers identified except two are between islands, indicating that salt water may function as an important barrier to the dispersal of the Andaman keelback.

While most barriers are intuitive, there are some caveats. The barrier between South Andaman (barrier a) and the rest of the northern islands (Fig. 7) could be due to lack of sampling points in the northern parts of South Andaman Island. Similarly, some of barriers detected ((d) in A, (e) in B, Fig. 7) could also be an artefact of lack of sampling in the central parts of the Middle Andaman Island.

### Vicariance and dispersal

Studies focusing on LGMs and the Pleistocene period have reconstructed maps to look at the influence of lowered levels of oceans on coastlines and vegetation patterns around the world. These maps clearly show that the Andamans was a continuous landmass during lowered sea levels (Voris, 2000; Ray & Adams, 2001; Woodruff, 2010). All the islands sampled in this study were connected during the late Pleistocene and were separated due to increases in sea level. Therefore, these shared haplotypes may have persisted in a large population when the Andaman landmass was continuous during the late (upper) Pleistocene (1,26,000–11,800 years ago) followed by rise in sea level which led to insularity of these islands. This insularity could have facilitated fixing of certain allelic forms of genes in smaller island populations.

As the shared haplotypes between islands do not always concur with their geographical proximity, other factors could have influenced gene flow. The Andaman Islands experience regular cyclones and strong monsoon winds which could facilitate over water dispersal. In addition, we cannot ignore the possibility of human mediated dispersal because there is a considerable amount of boat traffic and, historically, the Andamanese tribe are known to have used canoes to travel between islands (Radcliffe-Brown, 2013). Despite the vicariant history and several other factors which could enable gene flow, levels of haplotype diversity in mitochondrial genes of the Andaman keelback and their spatial segregation signals strong population structure, low dispersal abilities and barriers to dispersal.

## Conclusion

Considering the ever-increasing challenges for conserving diversity in the tropics, there is a critical need for studying the genetic diversity of populations and factors influencing the spatial distribution of diversity. This is even more pronounced for reptiles, as 95% of reptile species extinctions have occurred on islands (Frankham, Ballou & Briscoe, 2004). This study on the Andaman keelback provides insights into the limited gene flow between continental island populations of this species. The Andaman Islands were last connected during the LGM and rise in sea level has led to their insularity; nondisjunct distribution of the endemic fauna and flora on the Andaman Islands provides additional proof of its geological history.

Despite limitations in the number of markers and sampling, our study shows that populations of the Andaman keelback on the Andaman Islands are well differentiated, with reduced gene flow because of both IBD and salt water barriers to gene flow. While this pattern can be extrapolated to other terrestrial/freshwater reptiles in the Andaman Islands, we propose that future phylogeography studies can open avenues to comparative phylogeographic studies which are important to understand the impact of islands’ geological histories on contemporary population structures in combination with species specific traits.

## Supplemental Information

10.7717/peerj.5752/supp-1 Supplemental Information 1 Datasheet with accession numbers of genetic data and raw data. Sheet 1: Sample ID, corresponding gene, Bankit ID and sequence accession numbers; Sheet 2: Sample ID, population membership and population name; Sheet 3: Morphological data used for PCA analysis.

10.7717/peerj.5752/supp-2 Supplemental Information 2 AMOVA results. H 1 to H 7: labels of each haplotype. Number of ticks on the network between haplotypes correspond to number of base pair differences between them.

10.7717/peerj.5752/supp-3 Supplemental Information 3 Mantel Correlogram results. A: Populations identified by Geneland, B: Sex.

## Additional Information and Declarations

### Field Study Permissions

The following information was supplied relating to field study approvals (i.e., approving body and any reference numbers): Permission to conduct field surveys, including tissue collection from the Andaman keelback (listed under Schedule IV of the Indian Wildlife (protection) Act 1972) was obtained from the Department of Environment and Forests, Andaman and Nicobar Islands (Permit No. CWLW/WL/134/366). The permit from the Department of Environment and Forests covered all aspects of our study, including field survey, capture, morphological data collection and tail tip tissue collection.

### Data Availability

The following information was supplied regarding data availability: Raw data is provided in Supplemental File 1.
