# Supplementary material for: Population structure in the Andaman keelback, Xenochrophis tytleri: geographical distance and oceanic barriers to dispersal influence genetic divergence on the Andaman archipelago
Source: PeerJ. 2018 Oct 9;6:e5752. doi: 10.7717/peerj.5752 (PMC6183555; doi:10.7717/peerj.5752)
Supplement: Supplemental Information 2 — H 1 to H 7: labels of each haplotype. Number of ticks on the network between haplotypes correspond to number of base pair differences between them. [file peerj-06-5752-s002.pdf]

Run	No of groups	Details of groups	No of populations	Details of populations	Fst	Fsc	Fct	
1	3	1-SA, 2-LA, 3-MA,B,H,L,NA	7	Each island	0.82	0.54	0.61	
2	2	1-SA, LA 2-MA,NA,B,H,L	7	Each island	0.82	0.69	0.46	
2	3	1-SA, 2-LA, 3-MA,B,H,L,NA	4	1-SA,2-LA,3-MA,H,L,B 4-NA	0.77	0.23	0.7	
3	2	1-SA, LA 2-MA,NA,B,H,L	4	1-SA,2-LA,3-MA,H,L,B 4-NA	0.76	0.58	0.44	
		X	presented results					
		SA	South Andaman					
		LA	Little Andaman					
		MA	Middle Andaman					
		NA	North Andaman					
		B	Baratang					
		H	Havelock					
		L	Long Island					
