# Supplementary material for: Population structure in the Andaman keelback, Xenochrophis tytleri: geographical distance and oceanic barriers to dispersal influence genetic divergence on the Andaman archipelago
Source: PeerJ. 2018 Oct 9;6:e5752. doi: 10.7717/peerj.5752 (PMC6183555; doi:10.7717/peerj.5752)
Supplement: Supplemental Information 3 — A: Populations identified by Geneland, B: Sex. [file peerj-06-5752-s003.pdf]

Class Index	n dist	Mantel.cor	Pr(Mantel)	Pr(corrected)
D.cl.1 30km	5.26E+02	9.12E-02	0.001	0.001*
D.cl.2 70km	1.76E+03	4.29E-03	0.441	0.441
D.cl.3 130km	1.51E+03	-4.70E-01	0.001	0.003*
D.cl.4 240km	1.23E+03	-7.60E-02	0.01	0.020*
